# Synthesis and Enantioselective Pharmacokinetic/Pharmacodynamic Analysis of New CNS-Active Sulfamoylphenyl Carbamate Derivatives

**DOI:** 10.3390/ijms22073361

**Published:** 2021-03-25

**Authors:** Reem Odi, David Bibi, Bella Shusterman, Natalia Erenburg, Chanan Shaul, Claudiu T. Supuran, Alessio Nocentini, Meir Bialer

**Affiliations:** 1 Institute of Drug Research, School of Pharmacy, Faculty of Medicine, The Hebrew University of Jerusalem, Jerusalem 9112102, Israel; reem.odi@mail.huji.ac.il (R.O.); david.bibi1@mail.huji.ac.il (D.B.); bella.shusterman@mail.huji.ac.il (B.S.); natalia.erenburg@mail.huji.ac.il (N.E.); hananshaul@gmail.com (C.S.); 2 NEUROFARBA Department, University of Florence, Via Ugo Schiff 6, 50019 Sesto Fiorentino (Florence), Italy; claudiu.supuran@unifi.it; 3 David R. Bloom Center for Pharmacy, School of Pharmacy, Faculty of Medicine, The Hebrew University of Jerusalem, Jerusalem 9112102, Israel

**Keywords:** antiepileptic activity, carbonic anhydrase inhibition, CNS-active, carbamate, pharmacokinetics

## Abstract

We recently reported a new class of carbamate derivatives as anticonvulsants. Among these, 3-methylpentyl(4-sulfamoylphenyl)carbamate (MSPC) stood out as the most potent compound with ED_50_ values of 13 mg/kg (i.p.) and 28 mg/kg (p.o.) in the rat maximal electroshock test (MES). 3-Methylpropyl(4-sulfamoylphenyl)carbamate (MBPC), reported and characterized here, is an MSPC analogous compound with two less aliphatic carbon atoms in its structure. As both MSPC and MBPC are chiral compounds, here, we studied the carbonic anhydrase inhibitory and anticonvulsant action of both MBPC enantiomers in comparison to those of MSPC as well as their pharmacokinetic properties. Racemic-MBPC and its enantiomers showed anticonvulsant activity in the rat maximal electroshock (MES) test with ED_50_ values in the range of 19–39 mg/kg. (R)-MBPC had a 65% higher clearance than its enantiomer and, consequently, a lower plasma exposure (AUC) than (S)-MSBC and racemic-MSBC. Nevertheless, (S)-MBPC had a slightly better brain permeability than (R)-MBPC with a brain-to-plasma (AUC) ratio of 1.32 (S-enantiomer), 1.49 (racemate), and 1.27 (R-enantiomer). This may contribute to its better anticonvulsant-ED_50_ value. The clearance of MBPC enantiomers was more enantioselective than the brain permeability and MES-ED_50_ values, suggesting that their anticonvulsant activity might be due to multiple mechanisms of action.

## 1. Introduction

Out of 21 antiepileptic drugs (AEDs) introduced into the global market between 1989 and 2021, eleven are chiral molecules and nine of these AEDs were introduced into the market as a single enantiomer (or diastereomer). Only two chiral drugs (i.e., vigabatrin and fenfluramine) were introduced as racemates [1].

Two of the 21 AEDs contain a carbamate moiety (felbamate and cenobamate) in their chemical structure and two contain a sulfonamide (zonisamide) or a sulfamate (topiramate) moiety [2]. In addition, some new AEDs in development such as NBI-921352 (formerly XEN901), a selective inhibitor of Nav1.6 sodium channels, CVL-865, a selective positive allosteric modulator of GABAA, and bumetanide derivatives contain a sulfonamide in their chemical structure [3,4,5,6]. AND-287 is a chiral CNS-active sulfonamide derivative currently in development that its (R)-enantiomer demonstrated a more potent anticonvulsant activity than its (S)-enantiomer [1,7]. AND-287 has the following three structural elements: (a) an aryl portion that modulates potency, stability, and efficacy; (b) a benzyl substituent to increase the neuroprotective effect and bioavailability, and (c) a sulfamide moiety necessary to produce antiseizure and neuroprotective activity [7]. The anticonvulsant retigabine, currently being developed in a new oral formulation (XEN496), also contains a carbamate moiety in its molecular structure [3,4]. All the above shows that sulfonamide and carbamate moieties are of relevance in the development and design of new AEDs [8].

**Figure 1:**
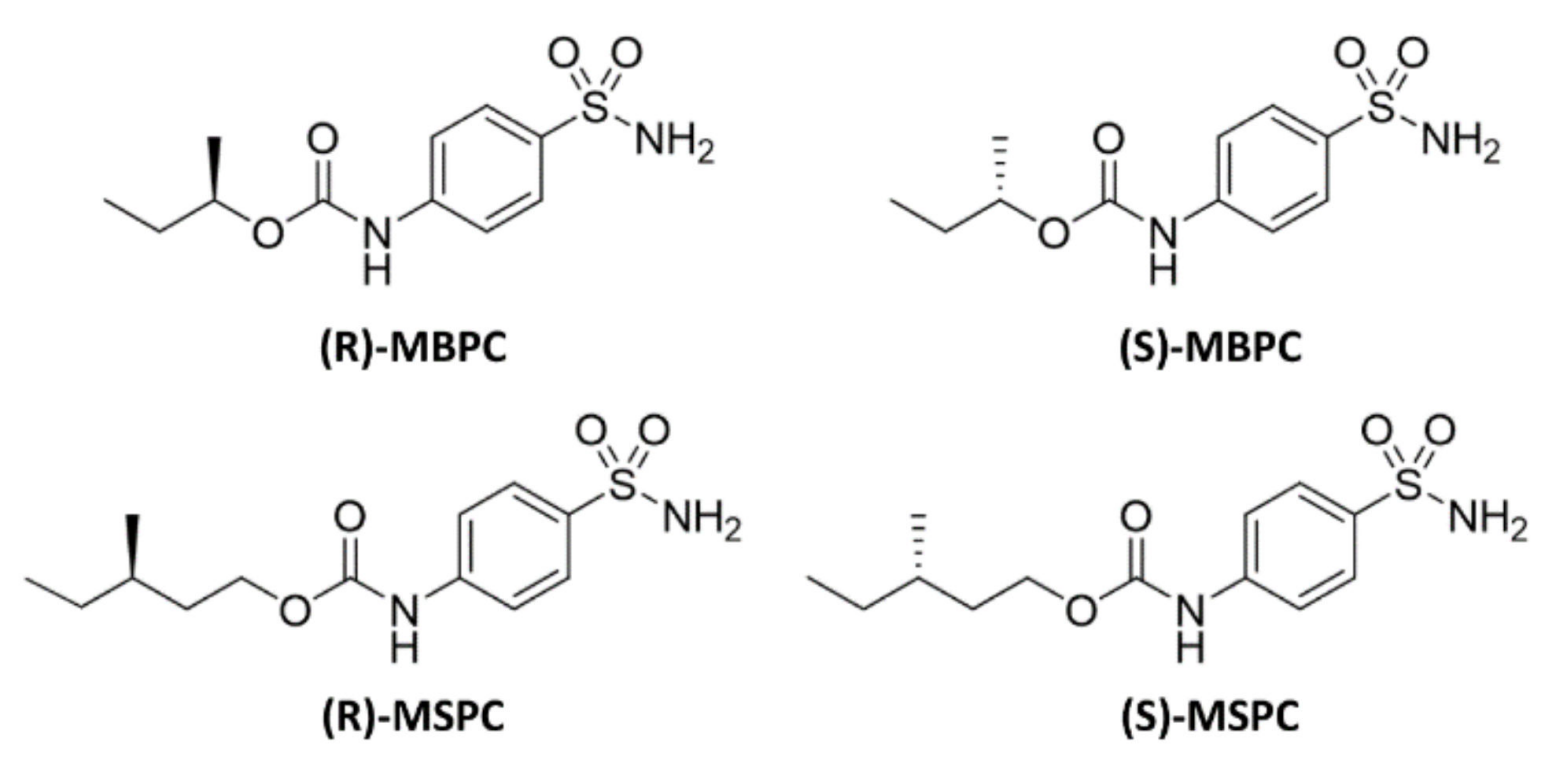
Chemical structures of the individual enantiomers of 3-methylpropyl(4-sulfamoylphenyl)carbamate (MBPC) and 3-methylpentyl(4-sulfamoylphenyl)carbamate (MSPC).

Lately, we reported a new class of (4-sulfamoylphenyl)carbamates as anticonvulsant derivatives. Among these, 3-methylpentyl(4-sulfamoylphenyl)carbamate (MSPC) showed the most potent effects in a maximal electroshock (MES) test with ED_50_ values of 13 mg/kg (i.p.) and 28 mg/kg (p.o.) in rats [9,10]. 3-Methylpropyl(4-sulfamoylphenyl)carbamate (MBPC) is an MSPC analogue with two less aliphatic carbons in its chemical structure (Figure 1). Both MSPC and MBPC have a stereogenic center, making it necessary to study both individual enantiomers for their anticonvulsant activity.

Indeed, drug chirality is a major issue in the design and development of new active pharmacological entities, since stereoisomers that possess the same chemical formula but different 3D conformations generally produce diverse pharmacological responses [11,12,13,14,15]. Many new chiral small molecule drugs were recently approved by the US Food and Drug Administration (FDA) as a single stereoisomer [13,16,17].

In this study, we report the synthesis of MBPC and assess the anticonvulsant activity, carbonic anhydrase (CA; EC 4.2.1.1) inhibition, and pharmacokinetics in rats of both MBPC enantiomers in comparison to MSPC and its enantiomers [9,10].

## 2. Results

**Scheme 1:**
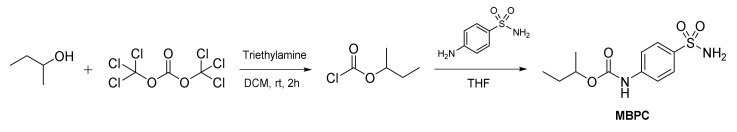
Synthesis of 3-methylpropyl(4-sulfamoylphenyl)carbamate (MBPC).

The synthesis of 3-methylpropyl (4-sulfamoylphenyl) carbamate (MBPC) and its single enantiomers was accomplished according to Scheme 1.

**Figure 2:**
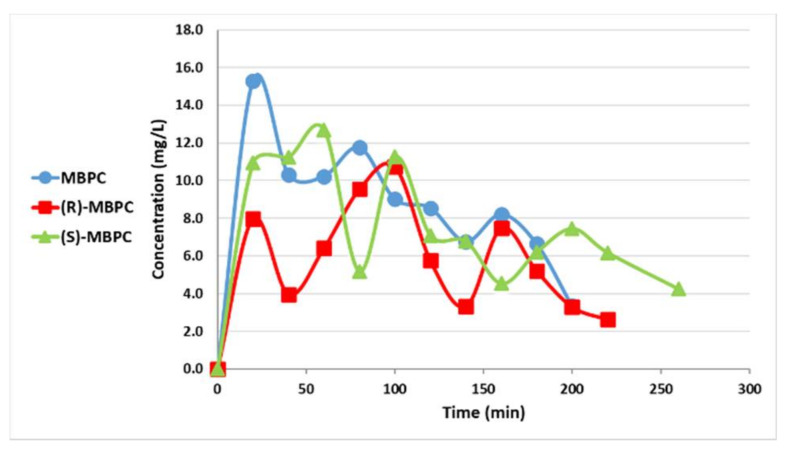
Plasma concentrations of 3-methylpropyl(4-sulfamoylphenyl)carbamate (MBPC) (racemate), (R)-MBPC, and (S)-MBPC after i.p. (80 mg/kg) administration to rats.

**Figure 3:**
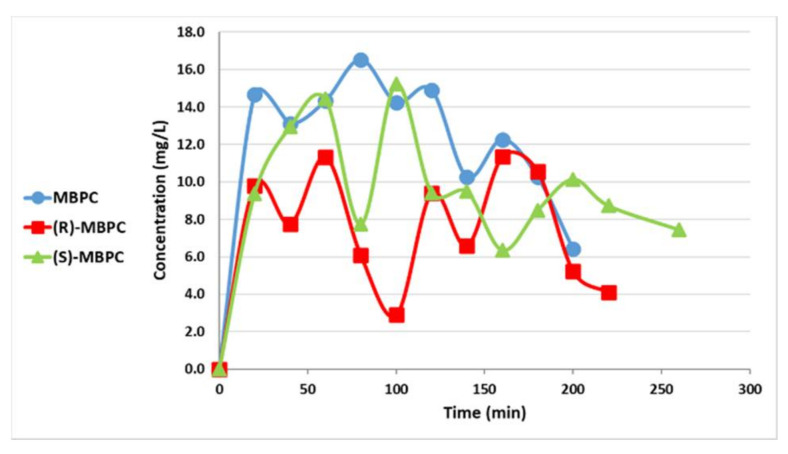
Brain concentrations of 3-methylpropyl(4-sulfamoylphenyl)carbamate (MBPC) (racemate), (R)-MBPC, and (S)-MBPC after i.p. (80 mg/kg) administration to rats.

The major pharmacokinetic (PK) parameters of racemic-MBPC and its individual enantiomers are depicted in Table 1 and Table 2. The mean plasma and brain concentrations of MBPC (racemate), (R)-MBPC, and (S)-MBPC after i.p. (80 mg/kg) administration to rats are illustrated in Figure 2 and Figure 3, respectively. As a result, the brain-to-plasma (AUC) ratio (BPR) of racemic-MBPC and its two individual enantiomers was as follows:

Racemic-MBPC = AUC_brain_/AUC_plasma_ = 48/32 = 1.49
(S)-MBPC = AUC_brain_/AUC_plasma_ = 52/40 = 1.32
(R)-MBPC = AUC_brain_/AUC_plasma_ = 30/24 = 1.27

**Table 1:** Mean pharmacokinetic (PK) parameters of 3-methylpropyl(4-sulfamoylphenyl)carbamate (MBPC) (racemate) and its two individual enantiomers calculated from plasma levels following i.p. (80 mg/kg dose) administration to rats.

PK Parameter	Racemic-MBPC	(R)-MBPC	(S)-MBPC
t_1/2_ (h)	0.5	0.6	1.2
Vss/F (L/kg)	4.3	6.7	5.7
CL/F (L/h/kg)	2.5	3.3	2.0
AUC_inf_ (mg/L/h)	32	24	39.6
C_max_ (mg/L)	15.3	10.7	12.7
t_max_ (h)	0.33	1.7	1
MRT (h)	1.7	2	2.7

**Table 2:** Mean pharmacokinetic (PK) parameters of 3-methylpropyl(4-sulfamoylphenyl)carbamate (MBPC) (racemate) and its two individual enantiomers calculated from brain levels following i.p. (80 mg/kg dose) administration to rats.

PK Parameter	Racemic-MBPC	(R)-MBPC	(S)-MBPC
t_1/2_ (h)	0.7	0.5	0.9
Vss/F (L/kg)	3.4	5.4	5
CL/F (L/h/kg)	1.7	2.6	1.6
AUC_inf_ (mg/L/h)	47.8	30.4	51.5
C_max_ (mg/L)	16.5	11.4	15.2
t_max_ (h)	1.33	2.7	1.66
MRT (h)	2	2.1	4.2
Brain-to-plasma (AUC_inf_) ratio	1.49	1.27	1.32

The rat-MES (p.o.) ED_50_ and TD_50_ values (and their 95% confidence interval—95% CI) of racemic-MBPC, (S)-MBPC, and (R)-MBPC in comparison to MSPC and its individual enantiomers, as well as their safety margin expressed as protective index (PI = TD_50_/ED_5__0_), are depicted in Table 3.

Table 4 shows the inhibition constants (K_I_s) of racemic-MBPC and its enantiomers, in comparison to MSPC and its enantiomers, against four human isoforms of carbonic anhydrase (hCA), namely the cytosolic and ubiquitous hCAs I and II, hCA IV (expressed in the CNS), and the brain-associated hCA VII. The compounds’ inhibitory potency spanned from a low to medium nanomolar range depending on the target hCA isoform. Racemic-MBPC and its enantiomers were medium potency hCA IV inhibitors but effectively inhibited hCAs I, II, and VII. (R)-MBPC was five times more effective than its (S)-enantiomer against hCA II, whereas (S)-MBPC was 2.9 and 3.7 times more effective than the (R)-enantiomer against hCAs I and VII, respectively.

## 3. Discussion

In 1992, the US Food and Drug Administration (FDA) issued a policy statement containing guidelines for the development of chiral drugs. In this document, the FDA recommends the evaluation of therapeutic utility and/or toxicological effects of each stereoisomer for chiral drug candidates [18]. Thus, there is an incentive to develop a chiral drug as a single individual enantiomer even if it is marketed first as a racemate [12,15]. In addition, a chiral switch from a racemate to one of its individual enantiomers is utilized to extend the period of patent exclusivity, since secondary pharmaceutical patents protect a range of aspects other than the active pharmaceutical ingredient (API-protected by the primary pharmaceutical patent) [15]. The term “chiral switch”, which is a component of secondary pharmaceutical patents, refers to the development of a single enantiomer from a chiral drug that was developed or marketed previously as a racemate. Thus, the essential criterion of a chiral switch is a change in the status of chirality [14]. The potential advantages of chiral switching are (a) improved therapeutic index through increased potency and selectivity; (b) a faster onset of action (e.g., (S)-ibuprofen); (c) lower susceptibility to drug–drug interactions, and (d) lower dosage compared to the racemate. It should be pointed out that a chiral switch is not clinically beneficial for all racemic drugs and sometimes offers little clinical advantage and is used by pharmaceutical manufacturers to perpetuate revenues as the original racemate approaches the end of its market exclusivity [13,19,20].

Since 1955, CNS-active alkylcarbamates have been developed for the treatment of epilepsy and other CNS disorders. Out of the regulatory approved carbamates, felbamate, retigabine, carisbamate, and cenobamate had different levels of success as AEDs. Carisbamate did not make it due to inconsistent clinical efficacy, and felbamate caused serious and sometimes life-threatening side effects that restricted its clinical use [9,10,21,22,23]. Cenobamate, approved by the FDA on November 2019, is currently showing potential for refractory patients with seizures that have been difficult to control with other AEDs [24,25].

MSPC was the most potent compound of a series of benzenesulfonamide alkylcarbamate derivatives reported lately [9]. MBPC was not included in such a series. A stereoselective PK and PD analysis showed that (R)-MBPC had a 65% higher clearance than its enantiomer and, consequently, a lower plasma exposure (AUC) than both (S)-MBPC and racemic-MBPC. Nevertheless, racemic-MBPC and its (S)- and (R)-enantiomers have the following brain-to-plasma (AUC) ratios (BPR) of 1.49, 1.32, and 1.27, respectively. The slightly better BPR of (S)-MBPC may contribute to its better rat anticonvulsant ED_50_ value. MBPC’s BPR values were better than that of (S)-MSPC but lower than those of racemic- and (R)-MSPC [10]. While (R)-MSPC has lower plasma and brain exposure compared to (S)-MSPC, the two MBPC enantiomers had similar brain permeability, with a slightly better BPR value for (S)-MBPC. The enantiostability of (R)- and (S)-MBPC was verified by extracting them from rat plasma and measuring their optical purity, and no change was observed in their specific optical rotation [α]_D_ value in comparison to their initial value when they were spiked in organic solvent prior to the optical rotation measurement. This shows that no chiral inversion was observed between the individual enantiomers of MBPC. Relevantly, a significant protective index was observed for MBPC and enantiomers as noted from the TD_50_ compared to the MES ED_50_ values reported in Table 3. A recently published study on similar benzenesulfonamide CAIs as antiepileptics showed no toxic effect toward the hepatic system associated with the use of this class of compounds [26].

Unlike MSPC, MBPC demonstrated enantiospecificity in the anticonvulsant activity of its individual enantiomers. The (S)-MBPC rat MES ED_50_ value was 19 mg/kg [95%CI = 13–25 mg/kg] compared to 39 mg/kg [95%CI = 27–51 mg/kg] for (R)-MBPC. This does not rule out the possibility that the anticonvulsant efficacy of MBPC enantiomers might be due to multiple mechanisms of action. It also demonstrates that a whole-body (in vivo) PD measure such as MBPC-MES-ED_50_ is enantioselective as an MBPC-specific PD measure, such as a CA inhibition K_I_ or the primary PK parameter clearance, which, in the case of MBPC, is mainly metabolic. The better anticonvulsant activity of (S)-MBPC is an incentive that it will be developed as a single individual enantiomer [12,15]. Nevertheless, this needs to be confirmed by other anticonvulsant models and does not rule out the possibility that both MBPC individual enantiomers might be candidates for further evaluation as potential new AEDs.

As previously suggested by us [9], the anticonvulsant activity of these classes of derivatives might be, in part, ascribed to the inhibition of human CAs which have been shown to be implicated in seizures. In fact, the human CNS is among the tissues/organs with the highest number of CA isoforms, among which are Cas I, II, III, IV, VA, VII, XII, and XIV [27]. Their inhibition has been exploited since the 1970s with new AEDs. Acetazolamide, topiramate, sulthiame, and zonisamide are CAI-marketed AEDs that contain a sulfonamide (or sulfamate) moiety in their chemical structure. The mechanisms by which CA isoforms possess antiepileptic activity is rather complex and there is no definitive consensus among researchers about this issue [27].

Racemic-MBPC showed an improved CA IV and VII inhibition potency compared to racemic-MSPC (K_I_ of 432 vs. 750 nM and 121 vs. 351 nM, respectively), whereas it acts as a weaker CA I and II inhibitor than its longer analogue (K_I_ of 97.1 vs. 77.0 nM and 20.2 vs. 7.6 nM, respectively). Of note, (R)-MBPC, as with (R)-MSPC, is the eutomer against CA II. In contrast, a eutomer swap occurred against CA I, with (S)-MBPC being (K_I_ of 71.8 nM) more active than (R)-MBPC (K_I_ of 203 nM). Additionally, the K_I_ increase in MBPC against CA IV accounts for the (S)-enantiomer as a eutomer, as in the case of MSPC. Interestingly, (S)-MBPC acted as a eutomer against CA VII (K_I_ of 53.3 nM), showing the greatest eutomer K_I_ increase with respect to MSPC.

Irrespective of the outcome of the sulfonamides and carbamates currently in development (e.g., NBI-921352, CVL-865) [28], alkyl- or aryl sulfonamides as well as sulfamoylphenyl carbamate derivatives will continue to represent an interesting class of compounds exhibiting anticonvulsant activity in animal models, coupled with a potential to help refractory epileptic patients.

## 4. Materials and Methods

### 4.1. Chemistry

All the solvents were of analytical grade or high-performance liquid chromatography (HPLC) grade and were purchased from Sigma-Aldrich, St. Louis, MI, USA.

#### 4.1.1. General Procedure for the Synthesis of Compounds

The general synthesis of of 3-methylpropyl(4-sulfamoylphenyl)carbamate (MBPC)) is described in Scheme 1. A solution of 2-butanol (for the synthesis of MBPC), (R)-2-butanol (for the synthesis of (R)-MBPC), or (S)-2-butanol (for the synthesis of (S)-MBPC) (1 equiv.) in dichloromethane (DCM, 5 mL) was added dropwise to a stirred solution of triphosgene (0.5 equiv.) and triethylamine (1 equiv.) in DCM (10 mL). The reaction mixture was allowed to stir for 2h at room temperature and then evaporated. The residue was dissolved in THF (10 mL). A solution of sulfanilamide (500 mg) in THF (10 mL) was added and stirred overnight. The reaction was quenched with water and extracted with EtOAc (15 mL). The organic layer was washed with water and brine, dried over sodium sulfate, filtered, and concentrated in vacuo. The desired product was obtained following flash chromatography (EtOAac/hexane). The identity of MBPC enantiomers and their purity were assessed by ^1^H NMR, HPLC, and elemental analysis.

#### 4.1.2. (R)- or (S)-3-Methylpropyl(4-sulfamoylphenyl)carbamate (MBPC)

^1^H NMR (300 MHz, DMSO-d6): δ 9.97 (s, 1H), 7.83–7.67 (m, 2H), 7.60 (d, *J* = 8.9 Hz, 2H), 7.21 (s, 2H), 4.74 (h, *J* = 6.4 Hz, 1H), 1.59 (pd, *J* = 7.3, 2.1 Hz, 2H), 1.23 (d, *J* = 6.2 Hz, 3H), 0.90 (t, *J* = 7.4 Hz, 3H).

Specific optical rotations [α]_D_^25^ of MBPC, (R)-MBPC, and (S)-MBPC in ethanol at room temperature were 0, −105, and +105 respectively, and their melting points were 187–191 °C.

### 4.2. Pharmacokinetics Studies

The pharmacokinetics (PK) of racemic-MBPC and its two individual enantiomers were studied following i.p. administration (80 mg/kg) to male Sprague Dawley rats weighing approximately 200–250 g, and their major PK parameters were estimated. The PK experiments were approved by the Ethical Committee of the Hebrew University’s Faculty of Medicine. The dose was administered to rats in multisol that is a mixture of propylene glycol, alcohol (EtOH), and water for injection at a ratio of 8:1:1. The study details were as described previously [29]. Plasma and brain levels of racemic-MBPC and its individual enantiomers were monitored at 20, 40, 60, 90, 120, 160, 180, 200, 220, and 260 min after dosing. Two rats were sacrificed at each time point.

### 4.3. Analysis of MBPC and Its Two Individual Enantiomers in Plasma and Brain

Plasma and brain concentrations of each compound were quantified by an HPLC assay. The HPLC analysis was performed on a system (2695 Separation Module; Waters, Milford, MA, USA) with a photodiode array UV detector (2996 PDA Detector; Waters, Milford, MA, USA) conditioned as follows: Kinetex, 5 u EVO C18 100 A, 1504.6-mm column (PhenomenexVR, Torrance, CA, USA). Linear gradients (5–95% acetonitrile content) with H_2_O (0.1% formic acid) and acetonitrile were used as the eluents with a flow rate of 1 mL/min at 20 °C. The compounds and the internal standard were detected at 250 nm. Plasma and brain concentrations of MBPC and its two individual enantiomers were quantified as previously described, [29].

### 4.4. Calculation of Pharmacokinetic (PK) Parameters

The PK parameters of each compound were calculated by non-compartmental analysis based on statistical moment theory using the PK software Phoenix Winnonlin Tripos L.P. (Pharsight Co., Mountain View, CA, USA) as previously described [30].

### 4.5. Anticonvulsant Activity of MBPC and Its Individual Enantiomers

The experiments with the MES model were conducted in male Sprague Dawley rats weighing 100–120 g (Charles River Laboratories, Wilmington, MA, USA) as previously described [30,31,32]. Eight rats were tested per dose in the dose–response curve in order to determine the ED_50_ value of each compound. The experiments were approved by the Ethical Committee of the NIH-ETSP program where the anticonvulsant activity was tested [30,31,32].

### 4.6. Carbonic Anhydrase Inhibition of MBPC and Its Individual Enantiomers

The carbonic anhydrase inhibition of MBPC and its individual enantiomers was assessed using a stopped-flow CO_2_ hydrase assay as previously described [33,34,35,36].

## Figures and Tables

**Table 3:** Quantitative anticonvulsant and minimal neurotoxicity data (ED_50_ and TD_50_ values) and protective indexes (PI) of 3-methylpropyl(4-sulfamoylphenyl)carbamate (MBPC) and 3-methylpentyl(4-sulfamoylphenyl)carbamate (MSPC). in rats dosed p.o. or i.p. ^b^.

Cmpd	MES-ED_50_ (mg/kg) [95% CI] ^a^	Neurotoxicity-TD_50_ (mg/kg) [95% CI] ^a^	Protective Index (PI = TD_50_/ED_50_)
Racemic-MBPC	25 [17–32]	>500	>20
(R)-MBPC	39 [27–51]	>500	>13
(S)-MBPC	19 [13–25]	>200	>11
Racemic-MSPC	28 [18–35]	>500	>18
Racemic-MSPC b	13 [8–22]	>250	>19
(R)-MSPC	30 [22–37]	NA	NA
(R)-MSPC b	39 [31–44]	134 [108–158]	3.4
(S)-MSPC	24 [17–29]	NA	NA
(S)-MSPC b	43 [33–52]	149 [121–157]	3.5

^a^. Median effective (ED_50_) and toxic (TD_50_) values. ^a^ In squared parentheses are the 95% confidence intervals (95%CI) determined by probit analysis. NA—not available. ^b^. Dosed i.p.

**Table 4:** Inhibition data of human carbonic anhydrase (CA) isoforms I, II, IV, and VII for of 3-methylpropyl(4-sulfamoylphenyl)carbamate (MBPC) and the standard sulfonamide inhibitor acetazolamide (AAZ) by a stopped-flow CO_2_ hydrase assay. The inhibition data previously reported for 3-methylpentyl(4-sulfamoylphenyl)carbamate (MSPC). are also shown.

Cmpd	K_I_ (nM) ^a^			
	CA I	CA II	CA IV	CA VII
MBPC	97.1 ± 5.3	20.2 ± 1.5	432 ± 28	121 ± 8
(S)-MBPC	71.8 ± 6.5	34.9 ± 1.4	249 ± 13	53.3 ± 2.9
(R)-MBPC	203 ± 13	7.1 ± 0.4	522 ± 26	197 ± 14
MSPC	77.0 ± 4.7	7.6 ± 0.5	750 ± 42	351 ± 26
(S)-MSPC	130 ± 8	20.3 ± 1.4	593 ± 42	744 ± 36
(R)-MSPC	34.1 ± 2.5	3.3 ± 0.2	810 ± 55	170 ± 9
AAZ	250 ± 15	12 ± 1	74 ± 5	5.7 ± 0.4

^a^. Inhibition data are expressed as means ± SEM of 3 different assays.
